# Whole genome sequencing and pan-genome analysis of *Staphylococcus/Mammaliicoccus* spp. isolated from diabetic foot ulcers and contralateral healthy skin of Algerian patients

**DOI:** 10.1186/s12866-023-03087-2

**Published:** 2023-11-16

**Authors:** Nerdjes Ferhaoui, Rina Tanaka, Tsuyoshi Sekizuka, Makoto Kuroda, Mohammed Sebaihia

**Affiliations:** 1 Laboratory of Molecular Biology, Genomics and Bioinformatics, Department of Biology, Faculty of Nature and Life Sciences, University Hassiba Benbouali, Chlef, Algeria; 2 Pathogen Genomics Center, National Institute of Infectious Diseases (NIID), Tokyo, Japan

**Keywords:** *Staphylococcus*, *Mammaliicoccus*, MRSA, Diabetic foot Ulcer Infection

## Abstract

**Background:**

Diabetic foot infections (DFIs) are the most common complications of diabetic foot ulcers (DFUs), and a significant cause of lower extremity amputation. In this study we used whole genome sequencing to characterize the clonal composition, virulence and resistance genetic determinants of 58 *Staphylococcus/Mammaliicoccus* spp. isolates from contralateral healthy skin and DFU from 44 hospitalized patients.

**Results:**

*S. aureus* (n = 32) and *S. epidermidis* (n = 10) isolates were recovered from both DFUs and healthy skin, whereas, *S. haemolyticus* (n = 8), *M. sciuri* (n = 1), *S. hominis* (n = 1) and *S. simulans* (n = 3) were recovered exclusively from healthy skin. In contrast, *S. caprae* (n = 2) and *S. saprophyticus* (n = 1) were recovered only from DFUs. Among *S. aureus* isolates, MRSA were present with high prevalence (27/32, 84.4%), 18 of which (66.7%) were from DFUs and 9 (33.3%) from healthy skin. In contrast, the coagulase-negative *Staphylococcus* (CoNS)/*Mammaliicoccus* isolates (n = 26), in particular *S. epidermidis* and *S. haemolyticus* were more prevalent in healthy skin, (10/26, 38.5%) and (8/26, 30.8%), respectively. MLST, spa and SCC*mec* typing classified the 32 *S. aureus* isolates into 6 STs, ST672, ST80, ST241, ST1, ST97, ST291 and 4 unknown STs (STNF); 8 spa types, t044, t037, t3841, t1247, t127, t639, t937 and t9432 and 2 SCC*mec* types, type IV and type III(A). Among CoNS, the *S. epidermidis* isolates belonged to ST54, ST35 and ST640. *S. haemolyticus* belonged to ST3, ST25, ST29, ST1 and ST56. The sole *M. sciuri* isolate was found to carry an SCC*mec* type III(A). A wide range of virulence genes and antimicrobial resistance genes were found among our isolates, with varying distribution between species or STs. The pan-genome analysis revealed a highly clonal population of *Staphylococcus* isolates, particularly among *S. aureus* isolates. Interestingly, the majority of *S. aureus* isolates including MRSA, recovered from the healthy skin and DFUs of the same patient belonged to the same clone and exhibited similar virulence/resistance genotype.

**Conclusions:**

Our study provides clinically relevant information on the population profile, virulence and antibiotic resistance of *Staphylococcus/Mammaliicoccus* spp. in DFIs, which could serve as a basis for further studies on these as well as other groups of pathogens associated with DFIs.

**Supplementary Information:**

The online version contains supplementary material available at 10.1186/s12866-023-03087-2.

## Background

Diabetes is a fast-growing global problem with huge social, health, and economic consequences [1]. The prevalence of diabetes in 2021 was estimated to be 10.5% (536.6 million people), and it is expected to raise to 12.2% (783.2 million) in 2045 [2].

People with diabetes are at increased risk of long-term complications such as coronary heart disease, cerebrovascular and peripheral vascular diseases and diabetic foot ulcers (DFUs) [3]. It was estimated that 15% of diabetics will develop DFUs during their existence [4].

Microbial infections of the DFUs, termed diabetic foot infections (DFIs) are key contributors to the amputation risk [5]. Limb loss associated with DFUs have a significant negative impact on mobility, psychosocial well-being, and quality of life of the patients and increase healthcare costs [6, 7].

Bacterial species belonging to the genus *Staphylococcus* are common colonizers of skin and mucous membranes of humans and animals, but also opportunistic pathogens capable of causing a wide range of infections. The staphylococci can be differentiated into Coagulase-Positive (CoPS) and Coagulase-Negative (CoNS), based on their ability to produce coagulase. Recently, five species among the CoNS (S. *sciuri, S. fleurettii, S. lentus, S. stepanovicii* and *S. vitulinus*), and belonging to *S. sciuri* group, were reclassified into the novel genus *Mammaliicoccus*, with *Mammaliicoccus* (*M*.) *sciuri* as the type species [8].

The CoPS, which are considered as more pathogenic than the CoNS, include the notorious pathogen, *S. aureus*, which is the main causative agent of both community acquired and nosocomial infections in humans as well as in animals [9–12], including DFIs [12, 13]. *S. aureus* can deploy numerous virulence factors which are implicated in DFI and delayed wound healing process [14, 15]. However, the CoNS and the M*ammaliicoccus* are now known to be also frequently associated with clinical infections, including diabetic foot osteomyelitis [16], particularly in immune-compromised and hospitalized patients [17].

Both *Staphylococcus* and *Mammaliicoccus* species are becoming increasingly resistant to several antibiotics, as a result of the acquisition of resistance genes [18], limiting further the therapeutic options against the infections caused by these bacteria, and leading to worse clinical outcomes [19].

Thus, understanding the genetic characteristics of *Staphylococcus* and related bacteria in DFIs can be exploited for both therapeutic and diagnostic purposes.

Apart from one study by Djahmi et al. (2013) [20], data on the staphylococci associated with DFIs in Algeria are lacking. Therefore, in the present study we used whole genome sequencing (WGS) to characterize the genetic diversity, antibiotic resistance and virulence genetic determinants of *Staphylococcus* and *Mammaliicoccus* spp. isolates recovered from DFIs; and to assess the potential association between clones/species and/or virulence/resistance genes with PEDIS grades and the source of isolates.

## Results

### Species and source of *Staphylococcus/Mammaliicoccus* isolates

Eight *Staphylococcus/Mammaliicoccus* species were identified among the 58 isolates, including *S. aureus* (32/58, 55.2%), *S. epidermidis* (10/58, 17.2%), *S. haemolyticus* (8/58, 13.8%), *S. simulans* (3/58, 5.2%), *S. caprae* (2/58, 3.5%), *S. hominis*, *S. saprophyticus* and *M. sciuri* (1/58 each, 1.7%). The characteristics of the 58 isolates included in this study are shown in Table 1.

**Table 1:** Clinical, epidemiological and molecular characteristics of *Staphylococcus/Mammaliicoccus* species recovered from DFUs and Healthy skin (n = 58)

Patient	Age/sex	PEDIS grade	Ulcer location	Antibiotics	Isolates (n)	Origin	species	SCC*mec*	ST-spa
P01	73/M	3	Forefoot	Ciprofloxacin	3	DFU	*S. aureus*	IVd(2B)	ST672-t3841
						HS	*S. aureus*	IVd(2B)	ST672-t3841
						HS	*S. epidermidis*	MSCoNS	ST54
P02	51/F	3	Midfoot	Amoxicillin-clavulanate	2	HS	*S. aureus*	IVc(2B)	ST80-t1247
						HS	*S. aureus*	III(3 A)	STNF-t037
P03	61/M	4	Heel	Metronidazole, Cefotaxime	3	DFU	*S.aureus*	IVc(2B)	ST80-t044
						HS	*S.aureus*	IVc(2B)	ST80-t044
						HS	*S. haemolyticus*	MSCoNS	ST3^§^
P04	62/M	3	Forefoot	Clindamycin, Ciprofloxacin	2	DFU	*S. epidermidis*	MSCoNS	ST54
						HS	*S. epidermidis*	MSCoNS	ST54
P05	60/F	4	Midfoot	Metronidazole, Cefotaxime	3	DFU	*S. epidermidis*	MSCoNS	ST54
						HS	*S. haemolyticus*	MSCoNS	ST29
						HS	*M. sciuri*	III(3 A)	STNF
P06	71/M	3	Forefoot	Ciprofloxacin	2	DFU	*S. epidermidis*	MSCoNS	ST54
						HS	*S. epidermidis*	MSCoNS	ST54
P07	61/M	3	Forefoot	Clindamycin	1	HS	*S. epidermidis*	MSCoNS	STNF
P08	63/F	3	Heel	Metronidazole, Cefotaxime	1	HS	*S. haemolyticus*	MSCoNS	ST25
P09	64/M	4	Forefoot	Cefotaxime	3	DFU	*S. aureus*	IVc(2B)	ST80-t044
						DFU	*S. caprae*	MSCoNS	STNF
						HS	*S. aureus*	IVc(2B)	ST80-t044
P10	73/M	2	Heel	Amoxicillin-clavulanate	2	DFU	*S. aureus*	MSSA	ST291-t937
						HS	*S. hominis*	MSCoNS	STNF
P11	66/M	2	Midfoot	Clindamycin	1	DFU	*S. aureus*	III(3 A)	STNF-t037
P13	54/M	4	Forefoot	Bactrim	1	HS	*S. epidermidis*	MSCoNS	ST35
P14	80/F	2	Forefoot	Imipenem	1	HS	*S. haemolyticus*	MSCoNS	ST3
P15	74/M	2	Heel	Amoxicillin-clavulanate	3	DFU	*S. aureus*	III(3 A)	ST241-t037
						HS	*S. aureus*	III(3 A)	ST241-t037
						HS	*S. epidermidis*	MSCoNS	ST35
P16	61/M	3	Forefoot	Cefazolin	2	DFU	*S. aureus*	III(3 A)	ST241-t037
						HS	*S. haemolyticus*	MSCoNS	ST3
P17	70/M	2	Heel	Bactrim	2	DFU	*S. epidermidis*	MSCoNS	ST640
						HS	*S. simulans*	MSCoNS	STNF
P18	49/M	4	Midfoot	Clindamycin, Cefotaxime	1	HS	*S. simulans*	MSCoNS	STNF
P19	44/M	3	Forefoot	Imipenem	2	DFU	*S. caprae*	MSCoNS	STNF
						HS	*S. simulans*	MSCoNS	STNF
P20	71/M	2	Midfoot	Imipenem	1	HS	*S. haemolyticus*	MSCoNS	ST1
P26	85/M	4	Heel	Clindamycin, Ciprofloxacin	1	DFU	*S. aureus*	III(3 A)	STNF-t037
P27	82/M	4	Forefoot	Metronidazole, Cefotaxime	1	HS	*S. haemolyticus*	MSCoNS	ST56
P28	72/M	4	Midfoot	Metronidazole, Cefotaxime	1	HS	*S. haemolyticus*	MSCoNS	ST25
P29	54/M	3	Heel	Bactrim, Cefotaxime	1	HS	*S. aureus*	IVc(2B)	ST80-t044
P30	59/M	3	Forefoot	Ciprofloxacin	1	HS	*S. aureus*	MSSA	ST97-t9432
P31	64/M	4	Forefoot	Clindamycin, Ciprofloxacin	1	DFU	*S. aureus*	IVd(2B)	ST672-t3841
P32	60/M	3	Heel	Metronidazole, Cefotaxime	2	DFU	*S. aureus*	III(3 A)	ST241-t037
						HS	*S. aureus*	III(3 A)	ST241-t037
S16*	57/F	3	Heel	Cefazolin	1	DFU	*S. aureus*	MSSA	STNF-t037
S17K*	72/M	2	Heel	Amoxicillin-clavulanate	1	DFU	*S. aureus*	IVc(2B)	ST80-t044
S104K*	60/F	2	Midfoot	Imipenem	1	DFU	*S. aureus*	IVc(2B)	ST80-t044
S4K*	59/M	2	Forefoot	Clindamycin, Ciprofloxacin	1	DFU	*S. saprophyticus*	MSCoNS	STNF
S6K*	49/M	3	Midfoot	Bactrim	1	DFU	*S. aureus*	IVc(2B)	ST80-t044
S7K*	58/M	3	Heel	Ciprofloxacin	1	DFU	*S. aureus*	IVc(2B)	ST80-t044
S8K*	60/M	4	Heel	Metronidazole, Cefotaxime	1	DFU	*S. aureus*	IVc(2B)	ST80-t044
S9K*	52/M	3	Midfoot	Cephalexin, Gentamicin	1	DFU	*S. aureus*	IVc(2B)	ST80-t044
S10K*	60/M	4	Forefoot	Metronidazole, Cefotaxime	1	DFU	*S. aureus*	IVc(2B)	ST80-t044
S11K*	67/M	4	Heel	Clindamycin, Ciprofloxacin	2	DFU	*S. aureus*	IVc(2B)	ST80-t044
			Forefoot			DFU	*S. aureus*	IVc(2B)	ST80-t044
S12K*	85/M	3	Forefoot	Ciprofloxacin	2	DFU	*S. aureus*	IVc(2B)	ST80-t044
			Heel			DFU	*S. aureus*	MSSA	ST1-t127
S14K*	60/F	4	Midfoot	Cefazolin	1	DFU	*S. aureus*	MSSA	ST1-t127

DFU: diabetic foot ulcer, HS: healthy skin; M: Male, F: female; MSSA: methicillin-sensitive S. aureus; MSCoNS: methicillin-sensitive coagulase negative staphylococci; ST: sequence type; STNF: ST not found; SCCmec: Staphylococcal Cassette Chromosome *mec*. *Patients sampled only from DFU (additional strains). § Novel allele, ST may indicate nearest ST. Values are numbers

Among the 32 *S. aureus* isolates, 23/32 (71.9%) were recovered from DFUs and 9/32 (28.1%) from healthy skin. The 10 *S. epidermidis* isolates were recovered from 8 patients, including 6/10 (60%) from healthy skin and 4/10 (40%) from DFUs. All the *S. haemolyticus* (n = 8), *M. sciuri* (n = 1), *S. hominis* (n = 1) and *S. simulans* (n = 3) isolates were recovered exclusively from healthy skin, but *S. caprae* (n = 2) and *S. saprophyticus* (n = 1) were recovered only from DFUs.

Among the 32 *S. aureus* isolates, 27/32 (84.4%) carried the *mecA* gene, and were therefore MRSA; 18/27 of which (66.7%) were from DFUs and 9/27 (33.3%) from healthy skin.

Statistically, *S. aureus* including MRSA isolates and CoNS were significantly associated with the source of isolation (*p*-value = 0.004).

### Molecular typing of the *Staphylococcus/Mammaliicoccus* isolates

Clones of *S. aureus* were characterized based on the combination of MLST, SCC*mec* and spa typing. *In silico* determination of MLST revealed that *S. aureus* isolates belonged to 6 known STs including ST80 (16/32, 50%), ST241 (5/32, 15.6%), ST672 (3/32, 9.4%), ST1 (2/32, 6.3%), ST97 and ST291 (1/32 each, 3.1%), and 4 STNF (4/32, 12.5%).

Eight spa types were identified among *S. aureus* isolates. The dominant one was t044 (13/32, 40.6%), followed by t037 (9/32, 28.1%), t3841 (3/32, 9.4%), t1247 and t127 (2/32 each, 6.3%), t639, t9432 and t937 (1/32 each, 3.1%).

Two SCC*mec* types were identified among MRSA isolates, type IV (19/27, 70.4%) and type III(A) (8/27, 29.6%). The type IV isolates were assigned to subtype IVc(2B) (16/27, 59.3%) and IVd(2B) (3/27, 11.11%).

Among CoNS/*Mammaliicoccus* isolates, SCC*mec* type III(A) (1/26, 3.8%) was detected in the sole *M. sciuri* isolate.

The dominant MRSA clone (13/27, 48.1%) was ST80- t044- IVc(2B), followed by ST241-t037- III(3 A)) (5/27,18.5%); whereas, ST672- t3841- IVd(2B) and STNF- t037- III(3 A) were each represented by 3/27 (11.1%) isolates. In addition, 2 other spa types were detected among ST80- IVc(2B) isolates, t1247 (2/27, 7.4%) and t639 (1/27, 3.7%).

2/5 (40%) of MSSA isolates belonged to ST1-t127, while ST97-t9432, ST291-t937 and STNF-t037 were each represented by one isolate (1/5, 20%).

Among *S. epidermidis* isolates, 6/10 (60%) belonged to ST54, 2/10 (20%) to ST35 and 1/10 (10%) to ST640. 3/8 (37.5%) *S. haemolyticus* belonged to ST3 and 2/8 (25%) to ST25, while ST29, ST1 and ST56 were each represented by 1/8 (12.5%) isolate.

### Virulence genes

The presence and distribution of the virulence genes are summarized in Tables 2,3 and S2.

A total of 116 virulence genes were detected among *S. aureus* isolates including 42 adhesion genes and a large number of type 8 capsular polysaccharide, immune evasion and exoenzyme genes.

**Table 2:** Virulence gene profiles in *S. aureus* isolates recovered from DFUs and Healthy skin (n = 32)

	ST1-t127 (n = 2) n(%)	ST241-t037 (n = 5) n(%)	ST291-t937 (n = 1) n(%)	ST672-t3841 (n = 3) n(%)	ST80-t044/t1247/t639 (n = 16) n(%)	ST97-t9432 (n = 1) n(%)	STNF-t037 (n = 4) n(%)	Total (n = 32) n(%)	^*§*^*p*-value
**Adhesion**									
*clfA*	2(100)	5(100)	0(0)	0(0)	0(0)	1(100)	4(100)	12(37.5)	< 0.001
*clfB*	2(100)	5(100)	0(0)	3(100)	0(0)	1(100)	2(50)	13(40.6)	< 0.001
*ebp*	2(100)	5(100)	1(100)	3(100)	16(100)	1(100)	4(100)	32(100)	N
*fnbA*	2(100)	0(0)	0(0)	0(0)	0(0)	0(0)	0(0)	2(6.3)	< 0.001
*fnbB*	2(100)	0(0)	0(0)	0(0)	1(6.3)	0(0)	0(0)	3(9.4)	0.002
*map*	0(0)	5(100)	0(0)	3(100)	15(93.8)	1(100)	4(100)	28(87.5)	0.001
*capA*	1(50)	0(0)	0(0)	1(33.3)	3(18.8)	1(100)	0(0)	6(18.8)	0.214
*cap8A*	2(100)	5(100)	1(100)	3(100)	16(100)	1(100)	4(100)	32(100)	N
*cap8B*	2(100)	5(100)	1(100)	3(100)	16(100)	1(100)	4(100)	32(100)	N
*cap8C*	2(100)	5(100)	1(100)	3(100)	16(100)	1(100)	4(100)	32(100)	N
*cap8D*	0(0)	5(100)	1(100)	3(100)	16(100)	1(100)	4(100)	30(93.8)	< 0.001
*cap8E*	0(0)	5(100)	1(100)	3(100)	16(100)	1(100)	4(100)	30(93.8)	< 0.001
*cap8F*	0(0)	5(100)	1(100)	3(100)	16(100)	1(100)	4(100)	30(93.8)	< 0.001
*cap8G*	0(0)	5(100)	1(100)	3(100)	16(100)	1(100)	4(100)	30(93.8)	< 0.001
*cap8H*	0(0)	5(100)	0(0)	3(100)	16(100)	0(0)	4(100)	28(87.5)	< 0.001
*cap8I*	2(100)	5(100)	0(0)	3(100)	16(100)	0(0)	4(100)	30(93.8)	< 0.001
*cap8J*	2(100)	5(100)	0(0)	3(100)	16(100)	0(0)	4(100)	30(93.8)	< 0.001
*cap8K*	2(100)	5(100)	0(0)	3(100)	16(100)	0(0)	4(100)	30(93.8)	< 0.001
*cap8L*	2(100)	5(100)	1(100)	3(100)	16(100)	1(100)	4(100)	32(100)	N
*cap8M*	2(100)	5(100)	1(100)	3(100)	16(100)	1(100)	4(100)	32(100)	N
*cap8N*	2(100)	5(100)	1(100)	3(100)	16(100)	1(100)	4(100)	32(100)	N
*cap8O*	2(100)	5(100)	1(100)	3(100)	16(100)	1(100)	4(100)	32(100)	N
*cap8P*	2(100)	5(100)	1(100)	3(100)	16(100)	1(100)	4(100)	32(100)	N
*capN*	1(50)	0(0)	0(0)	1(33.3)	3(18.8)	1(100)	0(0)	6(18.8)	0.214
*srtB*	2(100)	5(100)	1(100)	3(100)	15(93.8)	1(100)	4(100)	31(96.9)	0.984
*sdrC*	2(100)	5(100)	0(0)	1(33.3)	12(75)	1(100)	4(100)	25(78.1)	0.104
*sdrD*	2(100)	5(100)	0(0)	3(100)	15(93.8)	1(100)	4(100)	30(93.8)	0.014
*sdrE*	1(50)	3(60)	0(0)	0(0)	15(93.8)	1(100)	3(75)	23(71.9)	0.018
*icaA/B/C/D/R*	2(100)	5(100)	1(100)	3(100)	16(100)	1(100)	4(100)	32(100)	N
*isdA*	2(100)	5(100)	1(100)	3(100)	16(100)	1(100)	4(100)	32(100)	N
*isdB*	2(100)	5(100)	1(100)	3(100)	16(100)	1(100)	4(100)	32(100)	N
*isdC*	2(100)	5(100)	1(100)	3(100)	16(100)	1(100)	4(100)	32(100)	N
*isdD*	2(100)	5(100)	1(100)	3(100)	15(93.8)	1(100)	4(100)	31(96.9)	0.984
*isdE*	2(100)	5(100)	1(100)	3(100)	16(100)	1(100)	4(100)	32(100)	N
*isdF*	2(100)	5(100)	1(100)	3(100)	16(100)	1(100)	4(100)	32(100)	N
*isdG*	2(100)	5(100)	1(100)	3(100)	16(100)	1(100)	4(100)	32(100)	N
*isdI*	1(50)	0(0)	0(0)	1(33.3)	3(18.8)	1(100)	0(0)	6(18.8)	0.214
*harA*	1(50)	0(0)	0(0)	1(33.3)	3(18.8)	1(100)	0(0)	6(18.8)	0.214
Toxins									
Haemolysins									
*hly/hla*	2(100)	0(0)	1(100)	3(100)	16(100)	1(100)	0(0)	23(71.9)	< 0.001
*hlb*	1(50)	0(0)	1(100)	1(33.3)	5(31.3)	1(100)	0(0)	9(28.1)	0.161
*hld*	2(100)	5(100)	1(100)	3(100)	16(100)	1(100)	4(100)	32(100)	N
*hlgA/B/C*	2(100)	5(100)	1(100)	3(100)	16(100)	1(100)	4(100)	32(100)	N
Enterotoxins and Enterotoxin-like									
*sea*	2(100)	0(0)	0(0)	0(0)	0(0)	0(0)	4(100)	6(18.8)	< 0.001
*seb*	2(100)	0(0)	0(0)	0(0)	0(0)	1(100)	0(0)	3(9.4)	< 0.001
*seh*	2(100)	0(0)	0(0)	0(0)	0(0)	0(0)	0(0)	2(6.3)	< 0.001
egc cluster*	0(0)	0(0)	0(0)	3(100)	0(0)	0(0)	0(0)	3(9.4)	< 0.001
*sek*	2(100)	5(100)	0(0)	0(0)	0(0)	0(0)	4(100)	11(34.4)	< 0.001
*seq*	2(100)	5(100)	0(0)	0(0)	0(0)	0(0)	4(100)	11(34.4)	< 0.001
*selk*	2(100)	5(100)	0(0)	0(0)	0(0)	0(0)	4(100)	11(34.4)	< 0.001
*selq*	2(100)	5(100)	0(0)	0(0)	0(0)	0(0)	4(100)	11(34.4)	< 0.001
Staphylococcal exotoxin-like									
*set16*	1(50)	0(0)	0(0)	1(33.3)	0(0)	0(0)	0(0)	2(6.3)	0.060
*set17*	1(50)	0(0)	0(0)	1(33.3)	3(18.8)	1(100)	0(0)	6(18.8)	0.214
*set18*	1(50)	0(0)	0(0)	1(33.3)	3(18.8)	1(100)	0(0)	6(18.8)	0.214
*set19*	0(0)	0(0)	0(0)	1(33.3)	0(0)	1(100)	0(0)	2(6.3)	0.002
*set20*	1(50)	0(0)	0(0)	1(33.3)	3(18.8)	1(100)	0(0)	6(18.8)	0.214
*set21*	1(50)	0(0)	0(0)	0(0)	0(0)	0(0)	0(0)	1(3.1)	0.017
*set22*	1(50)	0(0)	0(0)	1(33.3)	3(18.8)	1(100)	0(0)	6(18.8)	0.214
*set23*	1(50)	0(0)	0(0)	1(33.3)	3(18.8)	1(100)	0(0)	6(18.8)	0.214
*set24*	1(50)	0(0)	0(0)	1(33.3)	3(18.8)	1(100)	0(0)	6(18.8)	0.214
*set25*	1(50)	0(0)	0(0)	1(33.3)	3(18.8)	1(100)	0(0)	6(18.8)	0.214
*set26*	1(50)	0(0)	0(0)	0(0)	0(0)	0(0)	0(0)	1(3.1)	0.017
Other toxins									
*edinB*	0(0)	0(0)	1(100)	0(0)	16(100)	0(0)	0(0)	17(53.1)	< 0.001
*lukS-PV/lukF-PV*	0(0)	0(0)	0(0)	0(0)	16(100)	0(0)	0(0)	16(50)	< 0.001
*lukD/E*	2(100)	5(100)	1(100)	3(100)	16(100)	1(100)	4(100)	32(100)	N
Type VII secretion system									
*esaA*	2(100)	5(100)	1(100)	3(100)	16(100)	1(100)	4(100)	32(100)	N
*esaB*	2(100)	5(100)	1(100)	3(100)	16(100)	1(100)	4(100)	32(100)	N
*esaD*	2(100)	0(0)	1(100)	3(100)	16(100)	1(100)	0(0)	23(71.9)	< 0.001
*esaE*	2(100)	0(0)	1(100)	3(100)	16(100)	1(100)	0(0)	23(71.9)	< 0.001
*esaG*	1(50)	0(0)	0(0)	1(33.3)	3(18.8)	1(100)	0(0)	6(18.8)	0.214
*esaG1*	1(50)	5(100)	1(100)	0(0)	0(0)	0(0)	4(100)	11(34.4)	< 0.001
*esaG2*	1(50)	0(0)	0(0)	2(66.7)	13(81.3)	0(0)	0(0)	16(50)	0.007
*esaG3*	1(50)	0(0)	0(0)	2(66.7)	0(0)	0(0)	0(0)	3(9.4)	0.006
*esaG4*	1(50)	5(100)	0(0)	2(66.7)	0(0)	0(0)	4(100)	12(37.5)	< 0.001
*esaG5*	1(50)	0(0)	0(0)	0(0)	0(0)	0(0)	0(0)	1(3.1)	0.017
*esaG6*	0(0)	0(0)	1(100)	2(66.7)	0(0)	0(0)	0(0)	3(9.4)	< 0.001
*esaG7*	1(50)	0(0)	0(0)	2(66.7)	0(0)	0(0)	0(0)	3(9.4)	0.006
*esaG8*	1(50)	0(0)	0(0)	0(0)	0(0)	0(0)	0(0)	1(3.1)	0.017
*esaG9*	1(50)	5(100)	0(0)	2(66.7)	13(81.3)	0(0)	4(100)	25(78.1)	0.091
*essA*	2(100)	5(100)	1(100)	3(100)	16(100)	1(100)	4(100)	32(100)	N
*essB*	2(100)	5(100)	1(100)	3(100)	16(100)	1(100)	4(100)	32(100)	N
*essC*	2(100)	0(0)	1(100)	3(100)	16(100)	1(100)	0(0)	23(71.9)	< 0.001
*esxA*	2(100)	5(100)	1(100)	3(100)	16(100)	1(100)	4(100)	32(100)	N
*esxB*	2(100)	0(0)	1(100)	3(100)	16(100)	1(100)	0(0)	23(71.9)	< 0.001
*esxC*	2(100)	0(0)	1(100)	3(100)	16(100)	1(100)	0(0)	23(71.9)	< 0.001
*esxD*	2(100)	0(0)	1(100)	3(100)	16(100)	1(100)	0(0)	23(71.9)	< 0.001
Degrading enzyme									
Lipases									
*geh*	2(100)	5(100)	1(100)	3(100)	16(100)	1(100)	4(100)	32(100)	N
*lip*	2(100)	5(100)	1(100)	3(100)	16(100)	1(100)	4(100)	32(100)	N
Proteases									
*sspA*	2(100)	5(100)	1(100)	3(100)	16(100)	1(100)	4(100)	32(100)	N
*sspB*	2(100)	5(100)	1(100)	3(100)	16(100)	1(100)	0(0)	28(87.5)	< 0.001
*sspC*	2(100)	5(100)	1(100)	3(100)	16(100)	1(100)	4(100)	32(100)	N
*splA*	2(100)	5(100)	1(100)	0(0)	16(100)	1(100)	4(100)	29(90.6)	< 0.001
*splB*	2(100)	5(100)	0(0)	0(0)	16(100)	1(100)	4(100)	28(87.5)	< 0.001
*splE*	2(100)	5(100)	1(100)	0(0)	0(0)	1(100)	4(100)	13(40.6)	< 0.001
Hyaluronidases									
*hysA*	2(100)	5(100)	1(100)	0(0)	16(100)	0(0)	4(100)	28(87.5)	< 0.001
Coagulases									
*coa*	2(100)	0(0)	1(100)	0(0)	0(0)	0(0)	0(0)	3(9.4)	< 0.001
*vWbp*	2(100)	5(100)	0(0)	0(0)	0(0)	0(0)	4(100)	11(34.4)	< 0.001
Other enzymes									
*aur*	2(100)	5(100)	1(100)	3(100)	16(100)	1(100)	4(100)	32(100)	N
Immune evasion									
*adsA*	2(100)	5(100)	1(100)	3(100)	16(100)	1(100)	4(100)	32(100)	N
*chp*	0(0)	5(100)	0(0)	0(0)	0(0)	0(0)	0(0)	5(15.6)	< 0.001
*sak*	2(100)	5(100)	0(0)	3(100)	14(87.5)	1(100)	4(100)	29(90.6)	0.077
*scn*	2(100)	5(100)	0(0)	3(100)	14(87.5)	1(100)	4(100)	29(90.6)	0.077
*spa*	2(100)	0(0)	1(100)	3(100)	16(100)	1(100)	0(0)	23(71.9)	< 0.001
*sbi*	2(100)	5(100)	1(100)	3(100)	16(100)	1(100)	4(100)	32(100)	N

^***§***^ *Chi-squared test was used to calculate p-values for describing the association of each gene and STs, p-values < 0.05 were considered statistically significant*

*egc cluster corresponds to seg, sei, sem, sen, seo and seu genes

*Values are numbers and percentages in brackets*

Thirty-five toxin-encoding genes were found among MRSA/MSSA isolates including 6 hemolysins (*hlgA, hlgB, hlgC, hlb, hld and hla/hly)*, 13 staphylococcal enterotoxins (*se*) and staphylococcal enterotoxin-like toxins (*sel*) (*sea, seb, seh, sek, seq, seg, sei, sem, sen, seo, seu selk and selq)*, with different carriage proportions ranging from 3.1 to 100%. ST1 isolates carried the highest number (n = 7) of *se/sel* genes, *sea, seb, seh, sek, seq, selk and selq*. In contrast, none of the *se/sel* genes were detected among the ST80 isolates.

Remarkably, none of the *Staphylococcus/Mammaliicoccus* isolates harbored an exfoliatine toxin gene (*eta* or *etb*) or a toxic shock syndrome toxin (*tst*) gene.

The leukocidins genes (*lukD, lukE)* were detected in all *S. aureus* isolates (32/32,100%), while *lukF/lukS-PV* were detected only in ST80 isolates (16/32, 50%). In addition, *edinB* gene encoding epidermal cell differentiation inhibitors was also detected only in ST80 isolates (16/32, 50%) and in ST291- t937 (1/32, 3.1%).

On the other hand, the sole virulence factor found among the 26 CoNS/*Mammaliicoccus* isolates was the arginine catabolic mobile element (*ACME*), which was exclusively present in *S. epidermidis* isolates (9/26, 34.6%).

**Table 3:** Virulence gene profiles in CoNS/*M. sciuri* isolates recovered from DFUs and Healthy skin (n = 26)

	*M.sciuri* (n = 1) n(%)	*S.caprae* (n = 2) n(%)	*S.epidermidis* (n = 10) n(%)	*S.haemolyticus* (n = 8) n(%)	*S.hominis* (n = 1) n(%)	*S.saprophyticus* (n = 1) n(%)	*S.simulans* (n = 3) n(%)	Total (n = 26) n(%)	*p-value
*ACME*	0(0)	0(0)	9(90)	0(0)	0(0)	0(0)	0(0)	9(34.6)	0.001

*Chi-squared test was used to calculate *p*-values for describing the association between genes and species, *p*-values < 0.05 were considered statistically significant

Values are numbers and percentages in brackets

The association of virulence genes with *S. aureus* ST or CoNS/*Mammaliicoccus* species was statistically significant. In contrast, no statistically significant association was found between the presence/absence of virulence genes and PEDIS grades or the source of isolates (*p*-value > 0.05).

### Resistance genes

#### *S. aureus*

The distribution of the genetic determinants of antibiotic resistance among the *S. aureus* is shown in Tables 4 and S2. Genes encoding β-lactam resistance, *mecA* and *blaZ*, were detected among *S. aureus* isolates at frequencies of 84.4% (27/32) and 50% (16/32), respectively.

**Table 4:** Resistance gene profiles in *S. aureus* isolates recovered from DFUs and Healthy skin (n = 32)

	ST1-t127 (n = 2) n(%)	ST241-t037 (n = 5) n(%)	ST291-t937 (n = 1) n(%)	ST672-t3841 (n = 3) n(%)	ST80-t044/t1247/t639 (n = 16) n(%)	ST97-t9432 (n = 1) n(%)	STNF-t037 (n = 4) n(%)	Total (n = 32) n(%)	*p-value		
β-lactams											
*mecA*	0(0)	5(100)	0(0)	3(100)	16(100)	0(0)	3(75)	27(84.4)	< 0.001		
*blaZ*	0(0)	5(100)	1(100)	3(100)	2(12.5)	1(100)	4(100)	16(50)	< 0.001		
*blaI*	0(0)	0(0)	0(0)	1(33.3)	0(0)	1(100)	0(0)	2(6.3)	0.002		
*blaR1*	0(0)	0(0)	0(0)	1(33.3)	0(0)	1(100)	0(0)	2(6.3)	0.002		
Aminoglycoside											
*ant(6)-Ia*	0(0)	0(0)	0(0)	0(0)	16(100)	1(100)	0(0)	17(53.1)	< 0.001		
*ant(9)-Ia*	0(0)	5(100)	0(0)	0(0)	0(0)	0(0)	0(0)	5(15.6)	< 0.001		
*aac(6’)- aph(2’’)*	0(0)	5(100)	0(0)	0(0)	0(0)	0(0)	3(75)	8(25)	< 0.001		
*aph(3’)-III*	0(0)	5(100)	0(0)	2(66.7)	13(81.3)	0(0)	0(0)	20(62.5)	0.005		
*aph(3’)-IIIa*	0(0)	0(0)	0(0)	1(33.3)	3(18.8)	1(100)	0(0)	5(15.6)	0.207		
sat4	0(0)	0(0)	0(0)	1(33.3)	3(18.8)	1(100)	0(0)	5(15.6)	0.207		
Macrolides											
*mph(C)*	0(0)	0(0)	0(0)	3(100)	0(0)	0(0)	0(0)	3(9.4)	< 0.001		
*msr(A)*	0(0)	0(0)	0(0)	3(100)	0(0)	0(0)	0(0)	3(9.4)	< 0.001		
*ermA*	0(0)	5(100)	0(0)	0(0)	0(0)	0(0)	0(0)	5(15.6)	< 0.001		
*ermC*	0(0)	0(0)	0(0)	0(0)	1(6.3)	1(100)	0(0)	2(6.3)	0.014		
Tetracycline											
*tet(K)*	0(0)	0(0)	0(0)	0(0)	2(12.5)	0(0)	0(0)	2(6.3)	0.907		
*tet(M)*	0(0)	5(100)	0(0)	0(0)	0(0)	0(0)	4(100)	9(28.1)	< 0.001		
*tet(38)*	1(50)	0(0)	0(0)	1(33.3)	3(18.8)	1(100)	0(0)	6(18.8)	0.214		
Fusidic acid											
*fusB*	0(0)	0(0)	0(0)	0(0)	2(12.5)	0(0)	0(0)	2(6.3)	0.907		
*fusC*	2(100)	0(0)	0(0)	0(0)	0(0)	0(0)	0(0)	2(6.3)	< 0.001		
Trimethoprim-sulfamethoxazole											
*dfrG*	0(0)	5(100)	0(0)	0(0)	0(0)	0(0)	0(0)	5(15.6)	< 0.001		
Fosfomycin											
*fosB*	0(0)	0(0)	0(0)	1(33.3)	0(0)	0(0)	0(0)	1(3.1)	0.126		

*Chi-squared test was used to calculate *p*-values for describing the association between genes and STs, *p*-values < 0.05 were considered statistically significant

Values are numbers and percentages in brackets

The genes encoding aminoglycoside-modifying enzymes (*AME*), *aph(3’)- III/ aph(3’)- IIIa*, were the most prevalent among *S. aureus* isolates (25/32, 78.1%). All ST80 isolates (16/32, 50%) were positive for *ant(6)-Ia* and *aph(3’)-III/ aph(3’)-IIIa* genes, and all ST241 isolates (5/32, 15.6%) were positive for 3 *AME* genes, *ant(9)-Ia, aac(6’)- aph(2’’)* and *aph(3’)- III.*

Four genes encoding resistance to macrolide-lincosamide-streptogramin B (MLSB) were detected in *S. aureus* isolates. The *msr(A)* and *mph(C)* genes were detected in all the ST672 isolates (3/32, 9.4%), and the *erm(A)* in all the ST241 isolates (5/32, 15.6%).

Three genes encoding resistance to tetracycline were detected among *S. aureus* isolates, *tet(M*) (9/32, 28.1%), *tet(K)* (2/32, 6.3%) and *tet(38)* (6/32, 18.8%). Remarkably, only isolates belonging to t037 and harboring *SCCmec*-III (ST241 and STNF) carried *tet(M*).

The *fusB* and *fusC* genes coding for fusidic acid resistance were detected in all the ST80-t1247 (2/32, 6.3%) and ST1-t127 (2/32, 6.3%), respectively. The *dfrG* gene coding for trimethoprim-sulfamethoxazole resistance was detected only in ST241 isolates (5/32, 15.6%).

#### CoNS/ *M. sciuri*

As presented in Table 5, various resistance genes were also identified among the twenty-six CoNS/ *M. sciuri* isolates. 18/26 (69.2%) carried both *mecA* and *blaZ* genes. Remarkably, the single *M. sciuri* isolate harbored both *mecA* and *mecA1* (1/1, 100%). 9/10 (90%) *S. epidermidis* isolates carried both *fusB* and *fosB* genes. In addition, *msr(A)* and *mph(C)* were detected in *S. epidermidis* (2/10, 20%) and *S. haemolyticus* (8/8,100%) isolates. Moreover, *ermC* was found in *S. haemolyticus* (5/8, 62.5%) and *S. epidermidis* (1/10, 10%) isolates. *aac(6’)- aph(2’’)*/ aac(*6’)-Ie/aph(2’’)-Ia* genes were detected in *S .haemolyticus* (7/8, 87.5%) and *M. sciuri* (1/1, 100%).

**Table 5:** Resistance gene profiles in CoNS species and *M. sciuri* recovered from DFUs and Healthy skin (n = 26)

	*M.sciuri* (n = 1) n(%)	*S.caprae* (n = 2) n(%)	*S.epidermidis* (n = 10) n(%)	*S.haemolyticus* (n = 8) n(%)	*S.hominis* (n = 1) n(%)	*S.saprophyticus* (n = 1) n(%)	*S.simulans* (n = 3) n(%)	Total (n = 26) n(%)	*p-value
Resistance genes									
β-lactams									
*blaZ*	0(0)	2(100)	10(100)	8(100)	1(100)	0(0)	0(0)	21(80.8)	< 0.001
*blaI*	0(0)	2(100)	1(10)	2(25)	0(0)	0(0)	0(0)	5(19.2)	0.103
*blaR1*	0(0)	1(50)	1(10)	2(25)	0(0)	0(0)	0(0)	4(15.4)	0.714
*mecA*	1(100)	0(0)	9(90)	8(100)	1(100)	0(0)	0(0)	19(73.1)	0.002
*mecA1*	1(100)	0(0)	0(0)	0(0)	0(0)	0(0)	0(0)	1(3.8)	< 0.001
*mecI*	1(100)	0(0)	1(10)	0(0)	0(0)	0(0)	0(0)	2(7.7)	0.038
*mecR1*	1(100)	0(0)	1(10)	0(0)	0(0)	0(0)	0(0)	2(7.7)	0.038
Aminoglycoside									
*aac(6’)- aph(2’’)*	0(0)	0(0)	0(0)	5(62.5)	0(0)	0(0)	0(0)	5(19.2)	0.030
*aac(6’)-Ie/aph(2’’)-Ia*	1(100)	0(0)	0(0)	2(25)	0(0)	0(0)	0(0)	3(11.5)	0.079
*aph(3’)-III*	0(0)	0(0)	0(0)	4(50)	0(0)	0(0)	0(0)	4(15.4)	0.100
*aph(3’)-IIIa*	0(0)	0(0)	0(0)	2(25)	0(0)	0(0)	0(0)	2(7.7)	0.560
*sat4*	0(0)	0(0)	0(0)	2(25)	0(0)	0(0)	0(0)	2(7.7)	0.560
Streptogramin									
*vat(B)*	0(0)	0(0)	0(0)	1(12.5)	0(0)	0(0)	0(0)	1(3.8)	0.886
*vat(C)*	0(0)	0(0)	0(0)	1(12.5)	0(0)	0(0)	0(0)	1(3.8)	0.886
*vgb(B)*	0(0)	0(0)	0(0)	1(12.5)	0(0)	0(0)	0(0)	1(3.8)	0.886
Macrolides									
*mph (C)*	0(0)	0(0)	2(20)	8(100)	0(0)	0(0)	0(0)	10(38.5)	0.004
*msr(A)*	0(0)	0(0)	2(20)	8(100)	1(100)	0(0)	0(0)	11(42.3)	0.003
*ermC*	0(0)	0(0)	1(10)	5(62.5)	0(0)	0(0)	0(0)	6(23.1)	0.110
*vga(A)*	0(0)	0(0)	0(0)	1(12.5)	0(0)	0(0)	3(100)	4(15.4)	0.004
*vga(B)*	0(0)	0(0)	0(0)	1(12.5)	0(0)	0(0)	0(0)	1(3.8)	0.886
*vga(A)LC*	0(0)	0(0)	0(0)	1(12.5)	0(0)	0(0)	0(0)	1(3.8)	0.886
*sal(A)*	1(100)	0(0)	0(0)	0(0)	0(0)	0(0)	0(0)	1(3.8)	< 0.001
Tetracycline									
*tet(L)*	0(0)	0(0)	0(0)	1(12.5)	0(0)	0(0)	0(0)	1(3.8)	0.886
*tet(K)*	0(0)	7(70)	0(0)	0(0)	0(0)	0(0)	0(0)	7(26.9)	0.018
Fusidic acid									
*fusB*	0(0)	1(50)	9(90)	3(37.5)	0(0)	0(0)	0(0)	13(50)	0.045
*fusC*	0(0)	0(0)	1(10)	1(12.5)	0(0)	0(0)	0(0)	2(7.7)	0.986
Trimethoprim-sulfamethoxazole									
*dfrG*	0(0)	0(0)	0(0)	5(62.5)	0(0)	0(0)	0(0)	5(19.2)	0.030
*dfrS1*	0(0)	0(0)	1(10)	0(0)	0(0)	0(0)	0(0)	1(3.8)	0.948
Fosfomycin									
*fosB*	0(0)	0(0)	10(100)	0(0)	0(0)	0(0)	0(0)	10(38.5)	< 0.001
Streptomycin									
*str*	0(0)	0(0)	0(0)	1(12.5)	0(0)	0(0)	0(0)	1(3.8)	0.886
kanamycin and neomycin									
*aadD*	0(0)	0(0)	0(0)	1(12.5)	0(0)	0(0)	0(0)	1(3.8)	0.886
Quaternary ammonium compounds									
*qacB*	0(0)	0(0)	0(0)	1(12.5)	0(0)	0(0)	0(0)	1(3.8)	0.886

*Chi-squared test was used to calculate p-values for describing the association between genes and species, p-values < 0.05 were considered statistically significant

*Values are numbers and percentages in brackets*

The *aph(3’)-*III*/ aph(3’)-IIIa* (6/8, 75%) and *tet(K)* (7/10, 70%) genes were found exclusively in *S. haemolyticus* and in *S. epidermidis*, respectively. Genes conferring resistance to streptogramin (*vat(B), vat(C)* and *vgb(B)*), macrolides (*vga(B)* and *vga(A)LC*), kanamycin/neomycin (*aadD), tetracycline* (*tet(L)*), streptomycin (*str*) and to quaternary ammonium compounds *(qacB*) were detected only in *S. haemolyticus* isolates, at a frequency of one gene per isolate(1/8, 12.5%).

### Pan-genome analysis

**Fig. 1:**
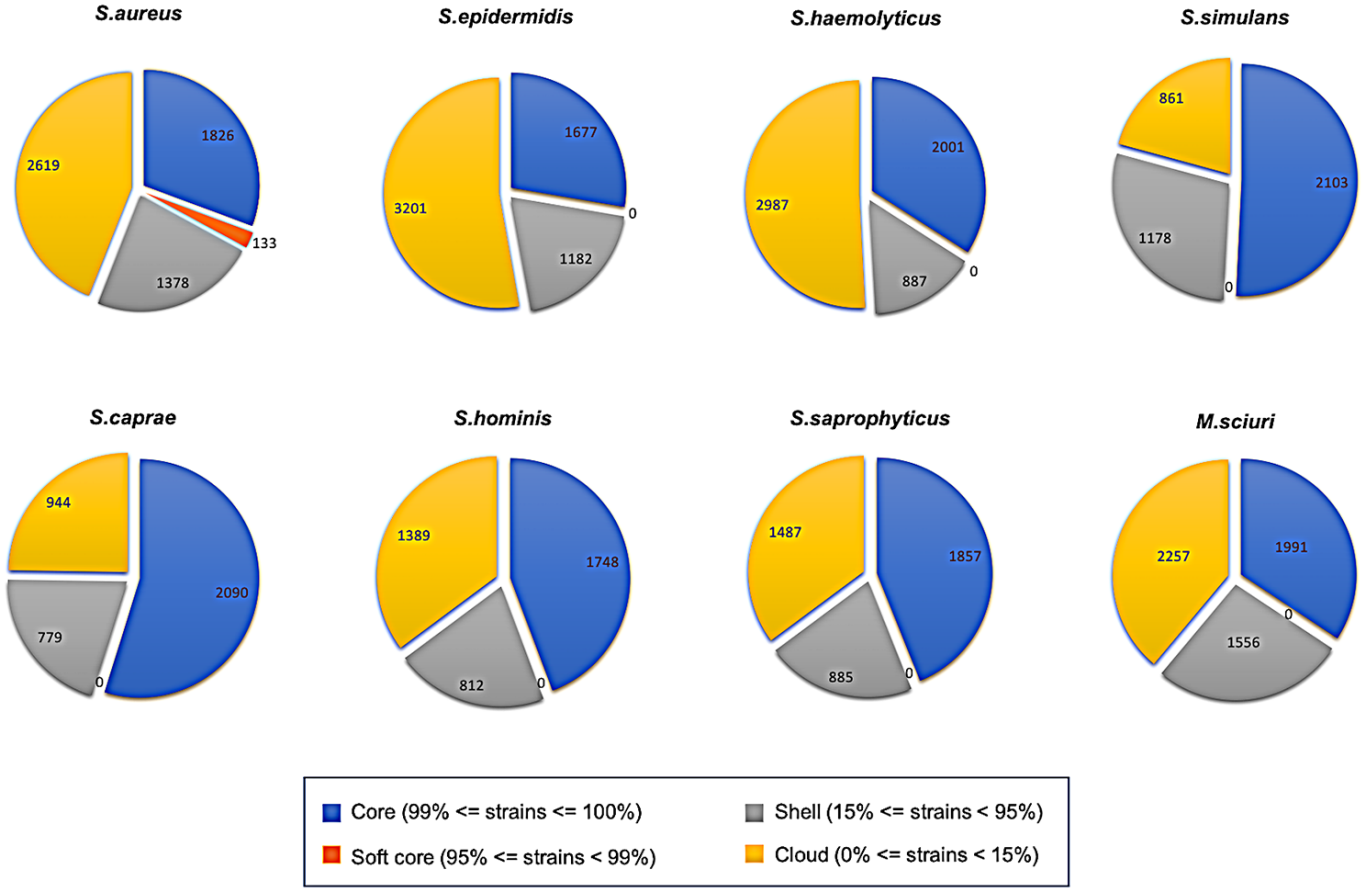
Pan-genome representation of *Staphylococcus/Mammaliicoccus* spp. The pan-genome pie charts show gene content of *Staphylococcus/Mammaliicoccus* spp., determined by the Roary software. The pan-genome can be classified into core genes (the combination of core and soft core genes) and accessory genes (the combination of shell and cloud genes)

The pan-genome of each species was determined and phylogenetic trees were built based on gene presence/absence matrix. Interestingly, isolates from DFUs are intermingled among the healthy skin isolates throughout the trees. A high number of accessory genes was observed, particularly, in *S. epidermidis* (4383, 72.3%), *S. aureus (*3997, 67.1%), *S. haemolyticus* (3874, 65.9%) and *M. sciuri* (3813, 65.7%) (Fig. 1).

**Fig. 2:**
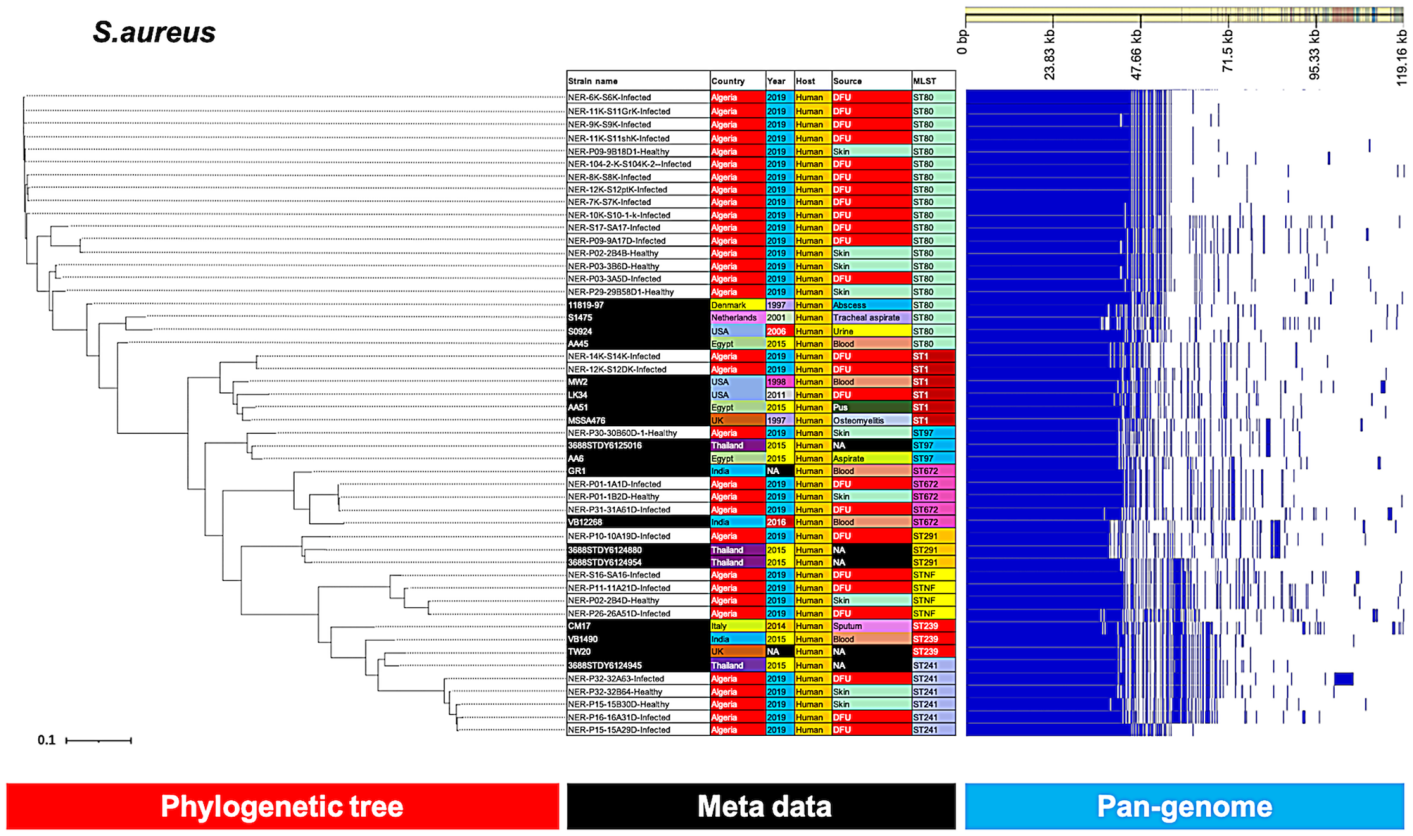
Phylogenetic analysis of *S. aureus* based on the pan-genomes with Roary. On the right, the heatmap was generated using the presence and absence of core and accessory genes produced by Roary, genes are represented by white and blue bar for absence and presence, respectively. The phylogenetic tree was visualized in the online interactive viewer Phandango using the absence and presence matrix of genes and the tree file in the standard Newick tree format generated by Roary. Meta data were shown in the middle, reference strains (11819-97, S1475, S0924, S0924, AA45, MW2, LK34, AA51, MSSA476, 3688STDY6125016, AA6, GR1, VB12268, 3688STDY6124880, 3688STDY6124954, CM17, TW20 VB1490, 3688STDY6124945) were highlighted by black in the strain name

The pan-genome analysis separated the ST80 *S. aureus* isolates into three subgroups (Fig. 2), one comprised of 10 closely related t044 isolates, the second comprised of 2 t1247 and 1 t639 related isolates, and the third contained 3 t044 isolates, which were more distantly related to ST80 strains from the other countries. *S. aureus* ST80, ST1, ST672, ST241 and STNF were found to be closely related to each other and were more distantly related to the reference strains.

**Fig. 3:**
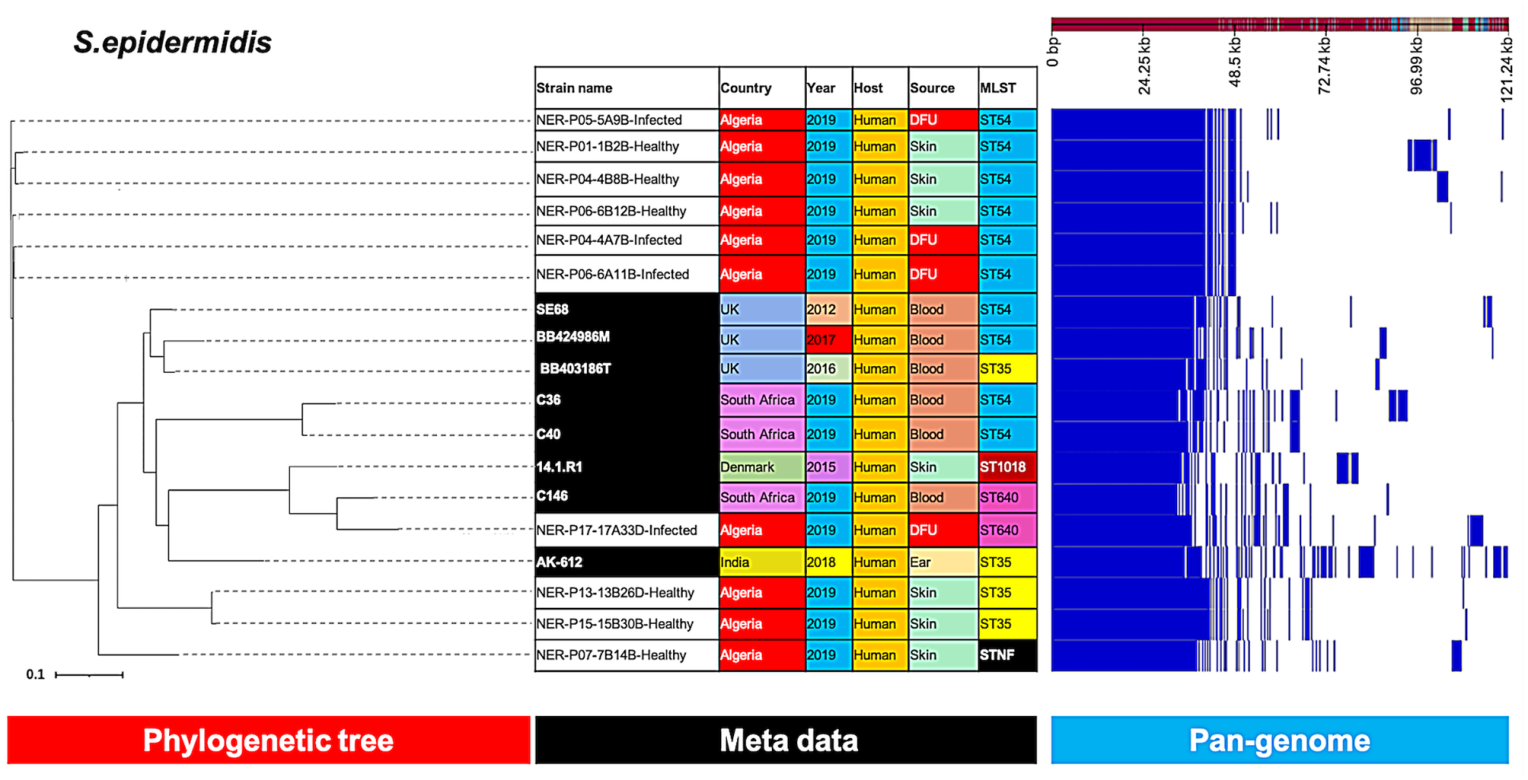
Phylogenetic analysis of *S. epidermidis* based on the pan-genomes with Roary. On the right of each panel, the heatmap was generated using the presence and absence of core and accessory genes produced by Roary, genes are represented by white and blue bar for absence and presence, respectively. Phylogenetic tree was visualized in the online interactive viewer Phandango using the absence and presence matrix of genes and the tree files in the standard Newick tree format generated by Roary. Meta data were shown in the middle, reference strains (SE68, BB424986M, BB403186T, C36, C40, 14.1.R1, C146, AK-612) were highlighted by black in the strain name

The phylogenetic tree of *S. epidermidis* revealed two major clusters, the first included ST54 isolates, which were distinct from the reference *S. epidermidis* strains, and the second included the reference ST54 strains (Fig. 3). The ST35 isolates clustered together, but separate from the ST35 strains from other countries.

**Fig. 4:**
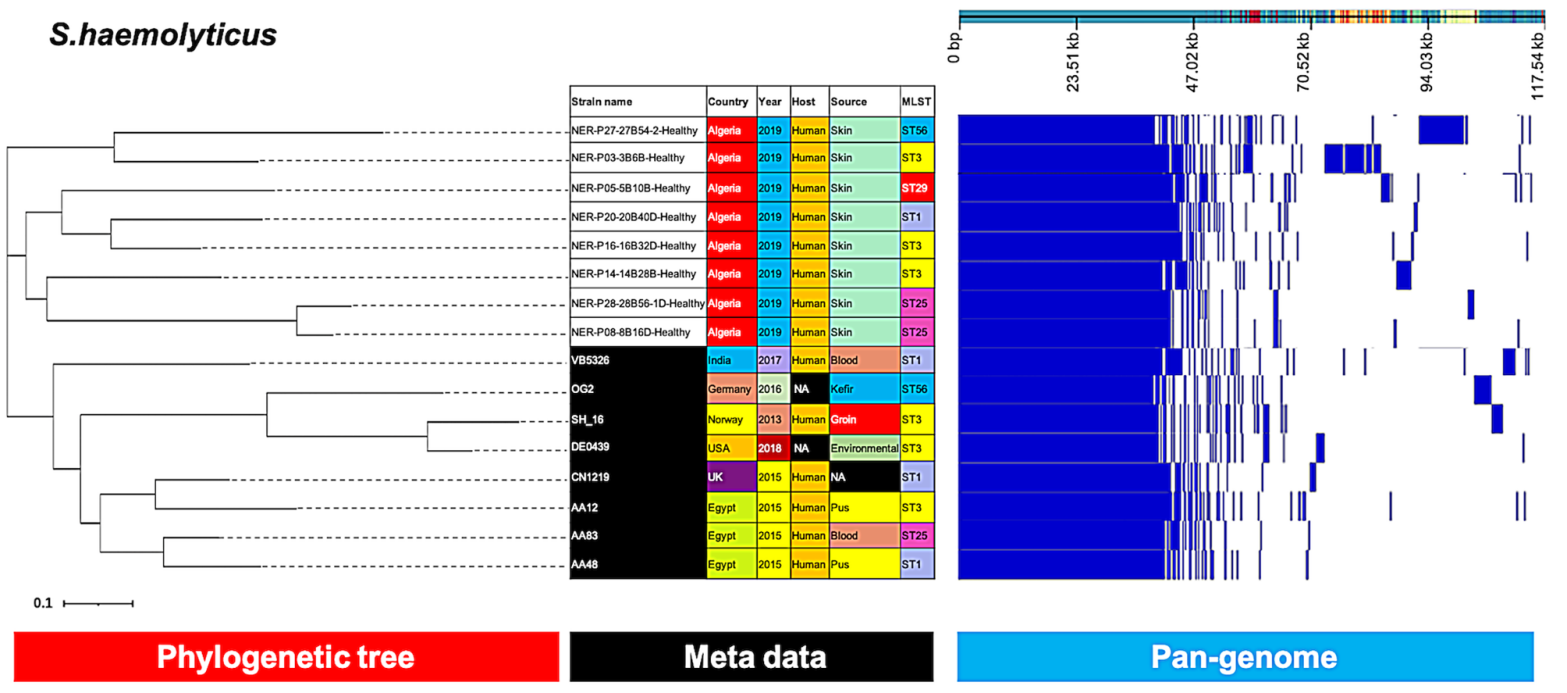
Phylogenetic analysis of *S. haemolyticus* based on the pan-genomes with Roary. On the right of each panel, the heatmap was generated using the presence and absence of core and accessory genes produced by Roary, genes are represented by white and blue bar for absence and presence, respectively. Phylogenetic tree was visualized in the online interactive viewer Phandango using the absence and presence matrix of genes and the tree files in the standard Newick tree format generated by Roary. Meta data were shown in the middle, reference strains (VB5326, OG2, SH_16, DE0439, CN1219, AA12, AA83, AA48) were highlighted by black in the strain name

The phylogenetic tree of *S. haemolyticus* revealed a clear distinction between our isolates and the reference strains (Fig. 4).

**Fig. 5:**
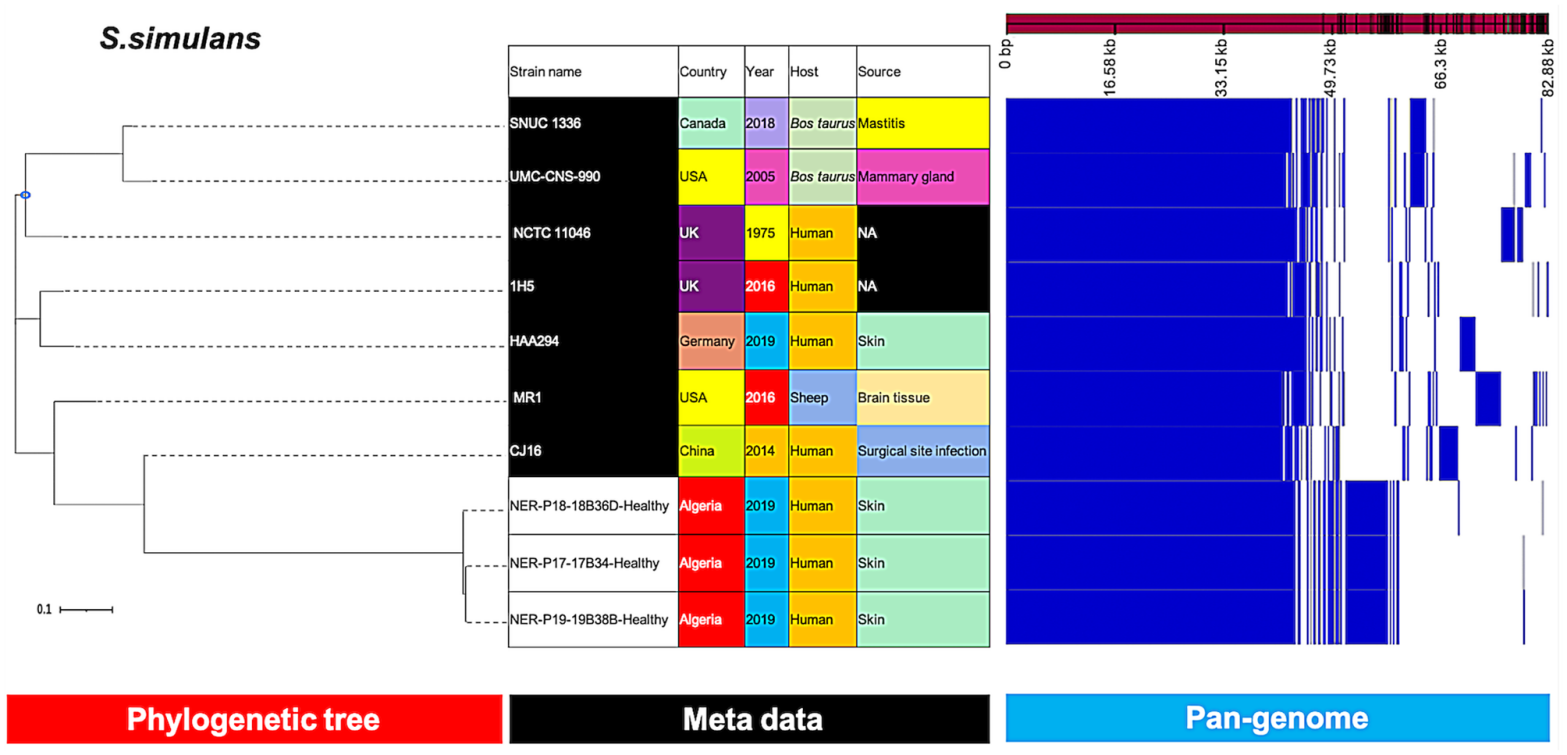
Phylogenetic analysis of *S. simulans* based on the pan-genomes with Roary. On the right of each panel, the heatmap was generated using the presence and absence of core and accessory genes produced by Roary, genes are represented by white and blue bar for absence and presence, respectively. Phylogenetic tree was visualized in the online interactive viewer Phandango using the absence and presence matrix of genes and the tree files in the standard Newick tree format generated by Roary. Meta data were shown in the middle, reference strains (SNUC 1336, UMC-CNS-990, NCTC 11,046, 1H5, HAA294, MR1, CJ16) were highlighted by black in the strain name

**Fig. 6:**
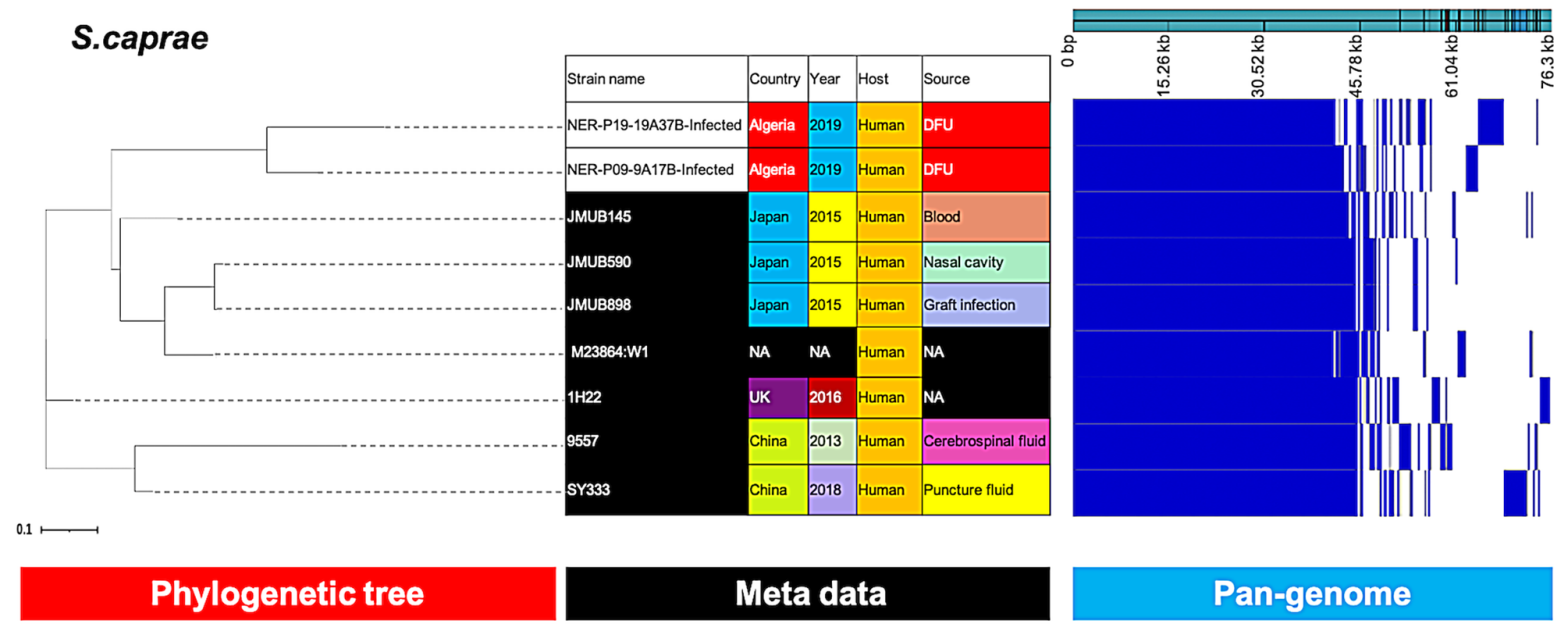
Phylogenetic analysis of *S. caprae* based on the pan-genomes with Roary. On the right of each panel, the heatmap was generated using the presence and absence of core and accessory genes produced by Roary, genes are represented by white and blue bar for absence and presence, respectively. Phylogenetic tree was visualized in the online interactive viewer Phandango using the absence and presence matrix of genes and the tree files in the standard Newick tree format generated by Roary. Meta data were shown in the middle, reference strains (JMUB145, JMUB590, JMUB898, M23864:W1, 1H22, 9557, SY333) were highlighted by black in the strain name

Similarly, *S. simulans* and *S. caprae* isolates (Figs. 5 and 6) from our study were closely related and more distantly related to the clinical isolates from China (CJ16) and Japan (JMUB145, JMUB590 and JMUB898), respectively.

**Fig. 7:**
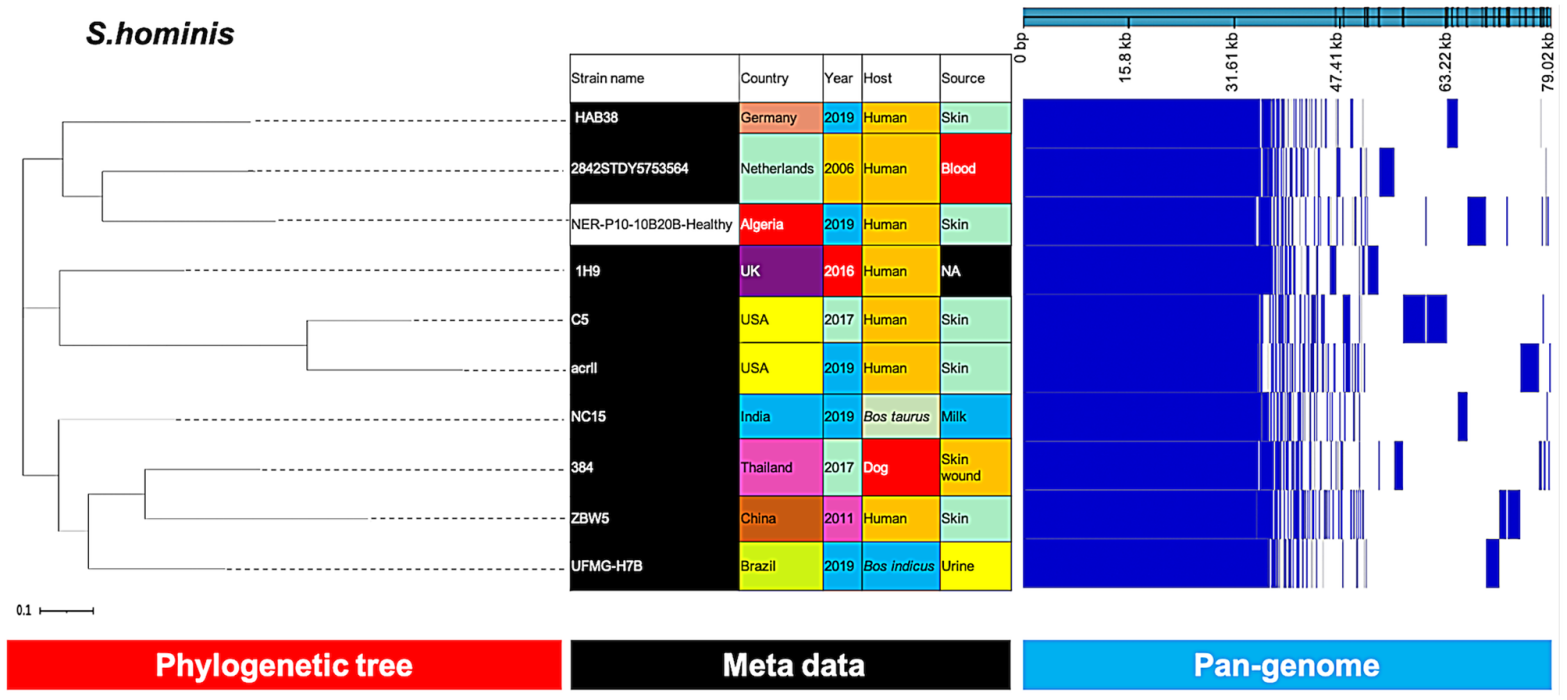
Phylogenetic analysis of *S. hominis* based on the pan-genomes with Roary. On the right of each panel, the heatmap was generated using the presence and absence of core and accessory genes produced by Roary, genes are represented by white and blue bar for absence and presence, respectively. Phylogenetic tree was visualized in the online interactive viewer Phandango using the absence and presence matrix of genes and the tree files in the standard Newick tree format generated by Roary. Meta data were shown in the middle, reference strains (HAB38, 2842STDY5753564, 1H9, C5, acrll, NC15, 384, ZBW5, UFMG-H7B) were highlighted by black in the strain name

**Fig. 8:**
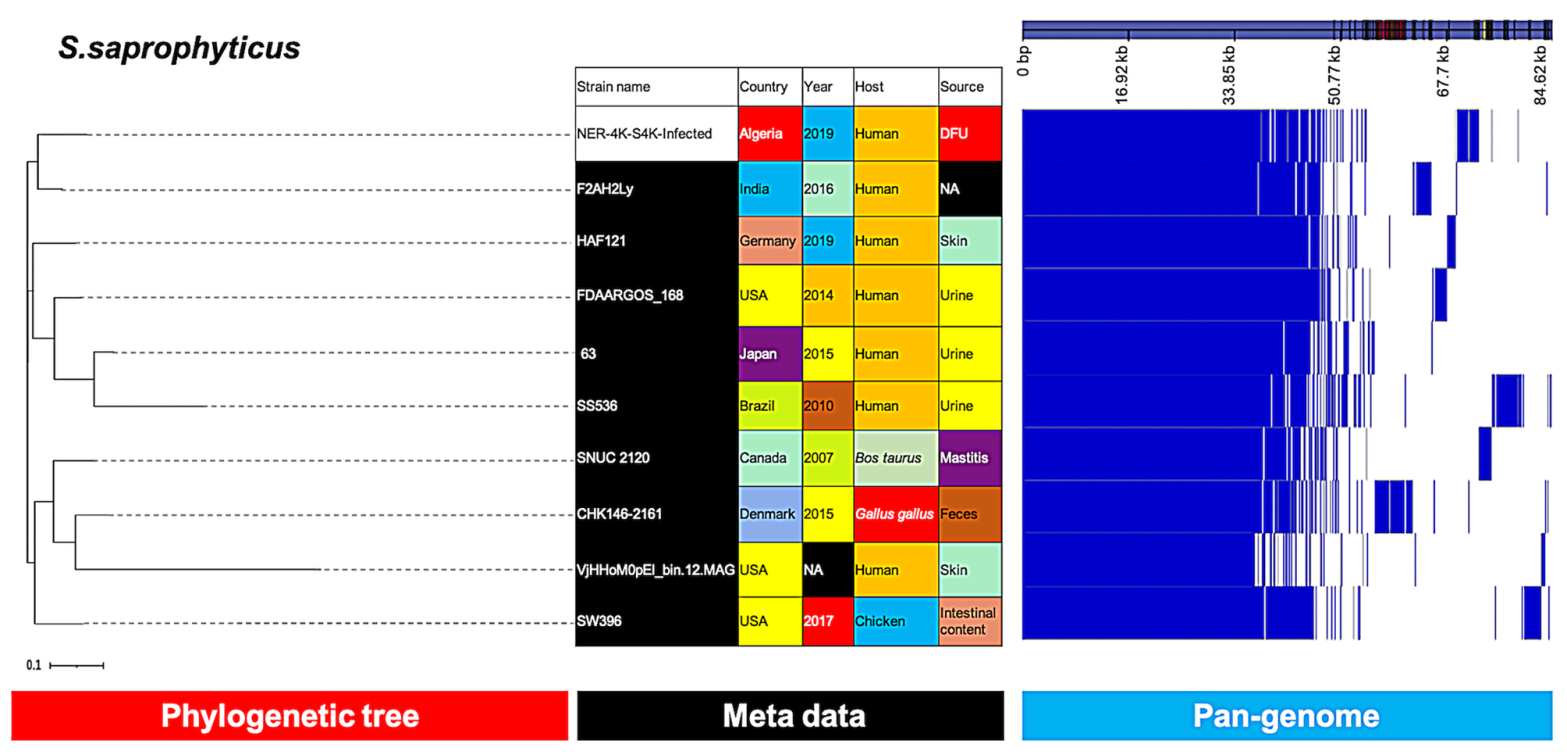
Phylogenetic analysis of *S. saprophyticus* based on the pan-genomes with Roary. On the right of each panel, the heatmap was generated using the presence and absence of core and accessory genes produced by Roary, genes are represented by white and blue bar for absence and presence, respectively. Phylogenetic tree was visualized in the online interactive viewer Phandango using the absence and presence matrix of genes and the tree files in the standard Newick tree format generated by Roary. Meta data were shown in the middle, reference strains (F2AH2Ly, HAF121, FDAARGOS_168, 63, SS536, SNUC 2120, CHK146-2161, VjHHoM0pEl_bin.12.MAG, SW396) were highlighted by black in the strain name

*S. saprophyticus* and *S. hominis* isolates clustered with clinical isolates from India and the Netherlands, respectively (Figs. 7 and 8).

**Fig. 9:**
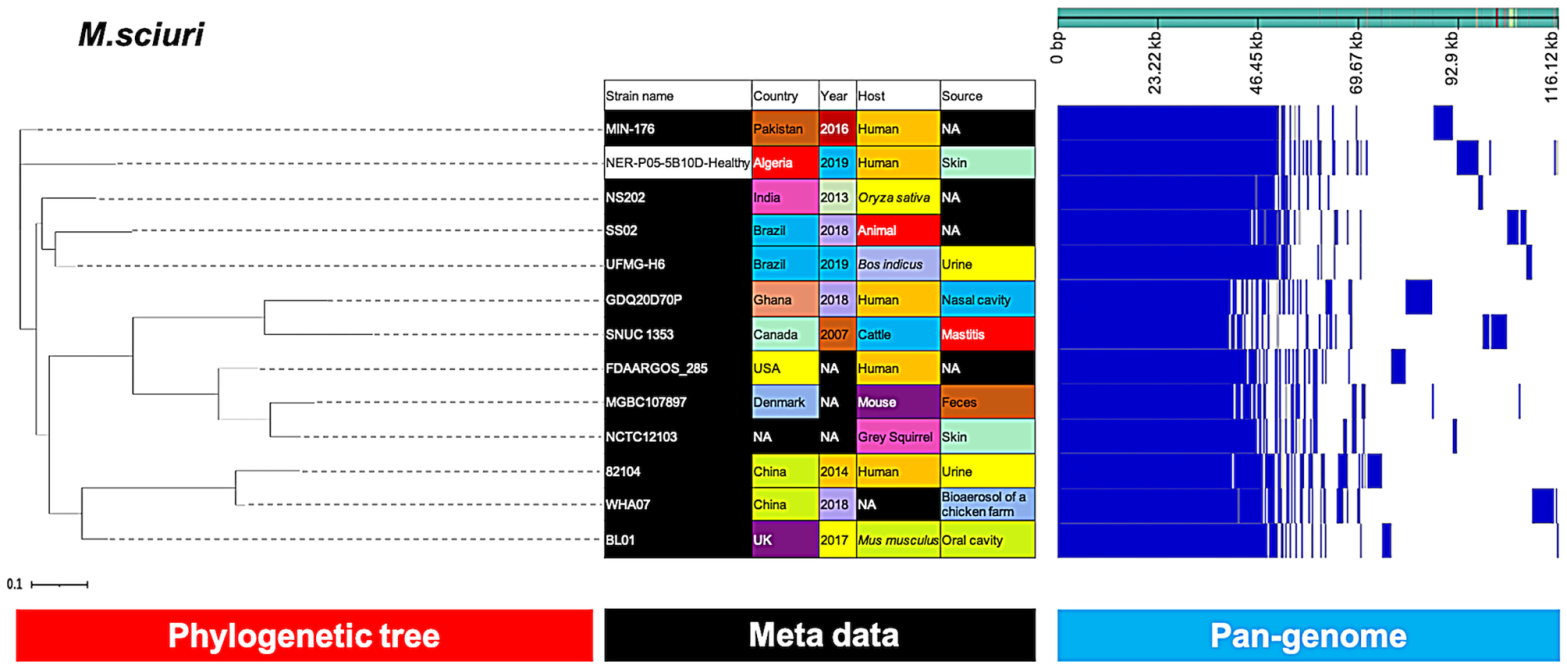
Phylogenetic analysis of *M. sciuri* based on the pan-genomes with Roary. On the right, the heatmap was generated using the presence and absence of core and accessory genes produced by Roary, genes are represented by white and blue bar for absence and presence, respectively. The phylogenetic tree was visualized in the online interactive viewer Phandango using the absence and presence matrix of genes and the tree file in the standard Newick tree format generated by Roary. Meta data were shown in the middle, reference strains (MIN-176, NS202, SS02, UFMG-H6, GDQ20D70P, SNUC 1353, FDAARGOS_285, MGBC107897, NCTC12103, 82,104, WHA07, BL01) were highlighted by black in the strain name

However, our single *M. sciuri* isolate formed an outgroup, which was distinct from the rest of the reference strains (Fig. 9).

## Discussion

In this study, we investigated clonal composition, virulence and resistance determinants of *Staphylococcus/Mammaliicoccus* species isolated from DFUs and healthy skin. *S. aureus* was recovered from the same sampling site alone or in combination with other CoNS/*Mammaliicoccus* species.

*S. aureus* isolates, including MRSA recovered from the healthy skin and DFUs of each patient, belonged mostly to the same clone and had similar genotype (P1, P3, P9, P15 and P32). This is consistent with earlier findings that *S. aureus* isolates recovered from 4 distinct anatomical sites (oro-nasal cavity, periodontal pockets, skin and ulcer) of patients with type 2 diabetes were highly related in the same patient [21]. However, MRSA isolates belonging to different clones were also recovered from the same sampling site (P2).

*S. aureus* isolates, in particular MRSA, were more prevalent in DFIs than the healthy skin. This finding led to suggest that *S. aureus*, especially MRSA play a significant role in the development and the chronicity of DFIs as described in previous studies [22, 23].

In contrast, CoNS species were mostly recovered from healthy skin, with the exception of *S. caprae* and *S. saprophyticus* which were isolated only from DFIs.

Diabetic patients are thought to be major vehicles for clonal dissemination of staphylococci between hospitals and the community [24]; this could explain the relatively high genetic diversity of our staphylococci. Indeed, our MRSA isolates carried SCC*mec* III or IV, which are commonly associated with healthcare- and community-acquired infections, respectively.

This study revealed that the dominant MRSA clone (13/27, 48.1%) had the characteristics of the European clone (ST80- t044- IVc(2B)) [25]. Only isolates belonging to this clone carried *lukF/lukS-PV.*

Despite the known community origin of *PVL*-positive ST80- IV [26], this clone was highly prevalent in our investigation, confirming the results of other studies from Algeria, which reported the widespread occurrence of *PVL*-positive ST80- IV in Algerian hospitals [27, 28]. Similarly, several studies have also reported that ST80 CA-MRSA was spreading in healthcare settings in Tunisia [29], Jordan [30] and Kuwait [31, 32].

In addition to *PVL*, isolates belonging to the European clone (ST80- t044- IVc(2B)) carried *edinB* and SCC*mec* type IV considered as stable genetic markers for CA-MRSA [26, 33].These properties suggest the community origin of this clone.

The EDIN coding genes are powerful molecular markers associated with poor wound outcomes, that could differentiate colonization from infection in DFUs [34, 35]. In our study, *edinB*-positive ST80- IVc(2B) isolates were recovered from different grades of severity (2–4), from both DFUs and the healthy skin, which indicates the lack of association between these virulence markers and the severity of DFUs. These results contrast with data obtained in France [34], where *edin* were found to be highly prevalent in *S. aureus* isolates from high-grade foot ulcers.

The Brazilian clone (ST241-t037- III(3 A)) was the second most prevalent clone in our study. This clone includes ST239, ST240 and ST241 harboring SCC*mec*-III(A), which differ in mutations in *pta* or *yqiL* genes [36].

Given that isolates belonging to the Brazilian clone (ST241-t037- III(3 A)) carried genes that confer resistance to several classes of antibiotics, corroborates a recent Algerian study performed in the province of Constantine that reported a high prevalence (72.5%) of a worrisome emerging multidrug resistant Brazilian clone (*PVL*-negative ST239/241 SCC*mec*-III mercury) [37]. Furthermore, this clone has been reported to be the major HA-MRSA clone in hospitals in another Algerian province [38].

Interestingly, another study from Algeria reported that 82.2% of the MRSA isolated from DFIs belonged to ST239 [20]. Furthermore, studies from India suggest that the Brazilian clone has been found to be associated with high biofilm production in DFUs, and positive for *luk-DE* and *icaA-B* [39].

ST672-t3841-IVd(2B) is another MRSA clone found in this study, it was detected among healthy skin and DFU isolates. ST672 is an emerging MRSA clone in India and Australia [40, 41] and commonly associated with CA-MRSA [42]. In addition, this clone has been reported in DFU patients in India [39]. To the best of our knowledge, this is the first report of this clone in Algeria.

The 5 MSSA isolates belonged to 4 different clones, ST1-t127 (2/5, 40%), ST97-t9432, ST291-t937 and STNF-t037 (1/5 each, 20%). The finding of ST1 among MSSA isolates was consistent with previous European studies [26, 43]. However, in the USA, this clone was reported as CA-MRSA, and was also found associated with DFIs across all healing categories [44].

Interestingly, two of our MSSA clones, ST291-t937 and ST97-t9432, were previously identified as livestock-associated [45]. ST291 was also reported in DFU patients in India [39]. We noted that the ST291-t937 isolate lacked the human innate immune evasion cluster (*IEC)* (*sak, chp, scn* and *sea)* which confirms its animal origin [46]. In contrast, the ST97-t9432 carried the *IEC*, harboring the *sak* and *scn* genes, which could suggest a human origin of this clone [47].

The phylogenetic analysis revealed that *Staphylococcus/Mammaliicoccus* spp. carried a high number of accessory genes which have features characteristic of transferred elements (presence of mobility genes) and may provide selective advantages under particular conditions such as antibiotic resistance, adaptation, colonization and pathogenicity [48].

Despite that certain strains clustered with refence strains, there was a clear distinction between our isolates and those from other countries. The phylogenetic comparison of ST80 with the European (11819-97 and S1475), Egyptian (AA45) and the USA (S0924) strains revealed that the Algerian ST80 strains were quite diverse from all the reference strains and mostly clonal, indicated by the extremely short branches at the tip of each clonal branch.

Both the MRSA- and MSSA-STNF exhibited a close clonality and slight variation in gene content, suggesting that the STNF-MRSA clone emerged following the acquisition of SCC*mec* [26]. In addition, the pan-genome analysis confirmed also that the STNF shared a common ancestor with ST241 and ST239.

*S. haemolyticus* isolates belonging to the same ST were not clustered together throughout the phylogenetic tree. Hence, STs that are intermingled with another may be a result of recent divergence or recombination of the MLST genes [49].

No statistically significant association was found between the presence of virulence genetic determinants and the severity of DFUs. This result contrasts with the findings of a previous report that suggested that infected DFU markers *sea, sei, lukE and hlgγ*, were strongly associated with strains from grades 2–4 DFUs, and that *cap8* was associated with strains from grade 1 ulcers and MSSA strains [50].

The higher frequency of adhesin-encoding genes among our *S. aureus* isolates suggests that they have a potential to form biofilms, which could contribute to their persistence and chronicity in DFU [39, 51].

Likewise, the higher prevalence of γ-hemolysins, *lukE-lukD* and *cap8* cluster genes among our *S. aureus* isolates was consistent with a previous study which reported high frequency of γ -hemolysin genes in MRSA isolates recovered from DFU specimens and patients nares [30].

The absence of *se/sel* genes in the ST80 clone, is in agreement with previous studies which found that ST80 CA-MRSA did not harbor any enterotoxin genes [29, 52]. However, a study in Kuwait hospitals reported that PVL- positive ST80 CA-MRSA carried *sed, sei, seg, seb, seh and sea*, suggesting that ST80 isolates arose from SE negative isolates due to the acquisition of SE-carrying bacteriophages [32].

In contrast, a high number of *se/sel* genes was found among ST672 and ST1 isolates. The production of a large number of superantigen exotoxins (Sag) might contribute to the worsening of DFUs by the activation of T cells and the production of proinflammatory cytokines [53].

The *ACME* was detected only in *S. epidermidis* isolates, which is in agreement with a previous study that reported a higher prevalence of *ACME* amongst *S. epidermidis* [54]. *ACME* contributes to the success of bacteria in acidic environments as the acid environment on the skin [55]. In fact, the extensive success of certain *S. aureus* strains, such as USA300 (ST8-MRSA-IVa), the US epidemic CA-MRSA strain, has been partially attributed to the presence of *ACME* which is thought to originate from *S. epidermidis* [56].

We have identified a wide range of antimicrobial resistance genes among our isolates, with varying distribution between species or ST. Genes coffering resistance to tetracycline, fusidic acid and fosfomycin were prevalent only in *S. epidermidis*. The macrolide resistance genes *erm(A)* and *erm(C)* were predominant in *S. aureus* ST241 clone and *S. haemolyticus*, respectively. Aouati et al. (2021) reported that *ermA* and *ermC* were responsible for erythromycin-resistance in multidrug resistant HA-MRSA ST239/241 strains in Algeria [37], which is in perfect agreement with our finding. Noteworthily, the *erm(A)* gene was previously reported as the most prevalent gene in MRSA strains in DFIs in Algeria [20].

Carriage of *AME* genes was mostly associated with *S. haemolyticus* and *S. aureus*, particularly ST80 and ST241 isolates, which proves that the monotherapy with AME fails to eradicate DFIs due to these bacteria.

Our data suggest a widespread distribution of resistant genes among *S. epidermidis* and *S. haemolyticus* isolates, the opportunistic pathogens which form part of the normal commensal flora of humans, whilst it is difficult to eradicate because of their resistance to multiple antibiotics [17]. In addition, *S. epidermidis* and other CoNS can provide a reservoir of genes facilitating MRSA infection such as antibiotic resistance determinants [11]. Thus, it has been suggested that *S. epidermidis* may play an essential role in DFI etiology [21].

The *tet(K)* and *fusB* genes were less abundant among ST80 isolates, which was in contrast with previous studies that demonstrated that Algerian PVL-positive ST80- IV strains were resistant to multiple antibiotics, in particular to these drugs [57–59].

Although our study provided some important information on the population and genetic profile of *Staphylococcus/Mammaliicoccus* spp. isolated from DFIs, it suffers from few limitations; (i) small sample size; (ii) lack of phenotypic antibiotic resistance data; and (iii) non-inclusion of a control group of patients who had not received antibiotics.

## Conclusions

In conclusion, our pan-genome analysis demonstrated that the Algerian *S. aureus* and CoNS/*Mammaliicoccus* isolates were closely related to each other, and presented novel genetic features, with a widespread distribution of virulence factors and antibiotic resistance genes, rendering this pathology more difficult to manage. The detection of the same *S. aureus/*CoNS clone in both DFIs and the healthy skin suggests that the autochthonous skin staphylococci can act as a reservoir for DFIs. To the best of our knowledge, this study represents the first investigation in Algeria, employing WGS and pan-genome analysis to get an insight on the underlying diversity and pathogenicity of *Staphylococcus/Mammaliicoccus* in DFIs. Most importantly, this study highlights the importance of WGS in disease surveillance and outbreak investigation, as it allows fine typing and detailed gene profiling of bacterial isolates.

## Methods

### Study group

Patients aged over 18 years who were hospitalized with infected DFUs at the University Regional Military Hospital and the University Hospital Ben Badis, in the province of Constantine, Algeria, from October to December 2019, were included in this study. 32 patients had a single ulcer and were sampled from both healthy foot skin (contralateral site to the chronic wound) and DFU. Patients who underwent surgical procedure including amputations were excluded from the study.

In addition, 14 strains obtained from the routine diagnostic recovered from 12 hospitalized DFU patients were added, including 2 patients who presented with 2 ulcers on the same foot.

DFUs were classified by clinicians using PEDIS classification (grade 2–4) proposed by the Diseases Society of America (IDSA) and the International Working Group on the Diabetic Foot Classifications of Diabetic Foot Infection (IWGDF) [60]. Patient demographics including age, gender, PEDIS grade, ulcer location and antibiotics taken during the 15 previous days were recorded.

### Sample collection

After wound debridement and cleansing with sterile saline solution, pus samples were collected in deep wounds from infected tissues. The healthy skin samples were obtained by swabbing of an intact skin area measuring 50 cm^2^. Swabs were immediately transported to the laboratory of microbiology in 1 ml of sterile saline 0.9% for culture. The strains were isolated on mannitol salt agar after incubation at 37 °C for 24 h.

### Whole genome sequencing analysis

Genomic DNA was extracted from 58 non duplicate *Staphylococcus/Mammaliicoccus* isolates by lytic treatment using achromopeptidase (Wako Pure Chemical Industries, Osaka, Japan) and then Sodium Dodecyl Sulfate (10%). DNA was purified using Zymo Research kit (Zymoresearch, Irvine, Ca, USA), according to the manufacturer’s instructions.

A DNA sequencing library (insert size, 300 to 500 bp) was prepared using a QIAseq FX DNA Library Kits (Qiagen, Germany). WGS was performed using the Illumina NextSeq 500 platform with the 300-cycle NextSeq 500 paired-end read sequencing (2 × 150-mer).

### Bioinformatic analysis

Annotation of the genomes was performed with Prokka [61]. Putative bacterial species were determined using Krona [62].

To characterize isolates, sequencing reads were analyzed *in silico* by multi locus sequence typing (MLST) [63]. spa types and SCC*mec* were identified *in silico* with the online tools spaTyper and SCC*mec*Finder [64]. Antimicrobial resistance genes were identified by homology searching against the ResFinder database [65].

For pan-genome analysis, 58 isolates from this study and publicly available sequences either at draft or complete genome sequences (a total of 78 strains) [see Additional file 3] was performed using Roary [66]. Tree construction was performed using FastTree and visualized in the online interactive viewer Phandango [67] using the absence and presence matrix of genes and the tree file in the standard Newick tree format generated by Roary.

### Statistical analysis

Data were analyzed using the Statistical Package for Social Sciences (SPSS; ver. 26.0). Contingency tables were constructed and Chi-squared tests were used to calculate *p*-value for describing possible associations between species/ST and virulence/resistance genes with PEDIS grades and the source of isolates. *p*-values < 0.05 were considered statistically significant.

### Electronic supplementary material

Below is the link to the electronic supplementary material.

Supplementary Material 1

Supplementary Material 2

Supplementary Material 3

## Data Availability

All data generated or analyzed in this study are included within the article and its additional files. The Short Read Archives (SRA) were deposited in NCBI database, [https://www.ncbi.nlm.nih.gov/bioproject?LinkName=sra_bioproject&from_uid=26401144] with the accession number PRJDB13730. All complete sequences in this study are available, as shown in the additional file 1.

## Declarations

### Laboratory of Molecular Biology, Genomics and Bioinformatics, Department of Biology, Faculty of Nature and Life Sciences, University Hassiba Benbouali, Chlef, Algeria

Nerdjes Ferhaoui and Mohammed Sebaihia.

### Pathogen Genomics Center, National Institute of Infectious Diseases (NIID), Tokyo, Japan

Makoto Kuroda, Tsuyoshi Sekizuka and Rina Tanaka.

### Nerdjes Ferhaoui

n.ferhaoui@univ-chlef.dz.

**Rina Tanaka**: r-tanaka@niid.go.jp.

### Tsuyoshi Sekizuka

tsuyoshi.sekizuka@gmail.com.

### Makoto Kuroda

makokuro@niid.go.jp.

## Abbreviations

CA-MRSA: Community-acquired MRSA
CoNS: Coagulase-negative *Staphylococcus*
CoPS: Coagulase-positive *Staphylococcus*
DFI: Diabetic foot infection
DFU: Diabetic foot ulcer
HA-MRSA: Healthcare-acquired MRSA
MRSA: Methicillin-resistant *S. aureus*
MSSA: Methicillin-sensitive *S. aureus*
MSCoNS: Methicillin-sensitive coagulase negative staphylococci
WGS: Whole genome sequencing
